# Case Report: An Anomalous Left Hepatic Venous Connection in a Patient With Unexpected Cyanosis

**DOI:** 10.3389/fped.2021.773935

**Published:** 2021-10-22

**Authors:** Fanyan Luo, Haisong Bu

**Affiliations:** Department of Cardiothoracic Surgery, Xiangya Hospital, Central South University, Changsha, China

**Keywords:** anomalous left hepatic venous connection, total cavopulmonary connection, cyanosis, surgery, intra-atrial tunnel

## Abstract

An anomalous left hepatic venous (LHV) connection is an extremely rare cardiac malformation, and left hepatic venous route abnormalities not associated with other cardiac lesions do not require surgical treatment because they are physiologically benign. However, when venous route abnormalities exist with associated cardiac lesions, the conduct of the cardiac surgical repair must accommodate the abnormal venous anatomy, especially in total cavopulmonary connection patients. Herein, we present a rare case of a 7-year-old Chinese boy about 1 year post bilateral superior vena cava pulmonary anastomosis who presented with severe cyanosis and was referred to our department. However, the patient showed an unexpected gradual decrease in blood oxygen saturation to 60–70% after the extracardiac total cavopulmonary connection (ETCPC) operation. Emergency echocardiography and computed tomography confirmed that the LHV entered the right atrium. Subsequently, the patient undergone completion of a staged TCPC with intra-atrial tunnel technique. This illustrative report highlights the essence of improving the preoperative accurate diagnosis to avoid unplanned reoperation in China, especially for the remote rural areas of eastern countries where the level of health care and services is relatively backward. Failure to identify anomalous LHV connection, in this case, will delay effective treatment past the optimal treatment time.

## Background

Anomalies of systemic venous connection (ASVC) are congenital disorders of the route or destination of the systemic venous return to the heart (1). ASVC can be either partial, involving part of the systemic venous return, or total, involving all of the systemic venous return. One hierarchical scheme for classification is based upon abnormalities of the inferior vena cava (IVC), the superior vena cava (SVC), and the hepatic veins (direct connection to the right atrium) (2, 3). In route abnormalities, the anomalous systemic venous return follows an abnormal pathway but eventually drains into the right-sided morphologically right atrium. Route abnormalities usually produce no physiologic sequelae. They become clinically important when they are associated with cardiac disorders requiring surgical repair (4).

## Case Presentation

**Figure 1:**
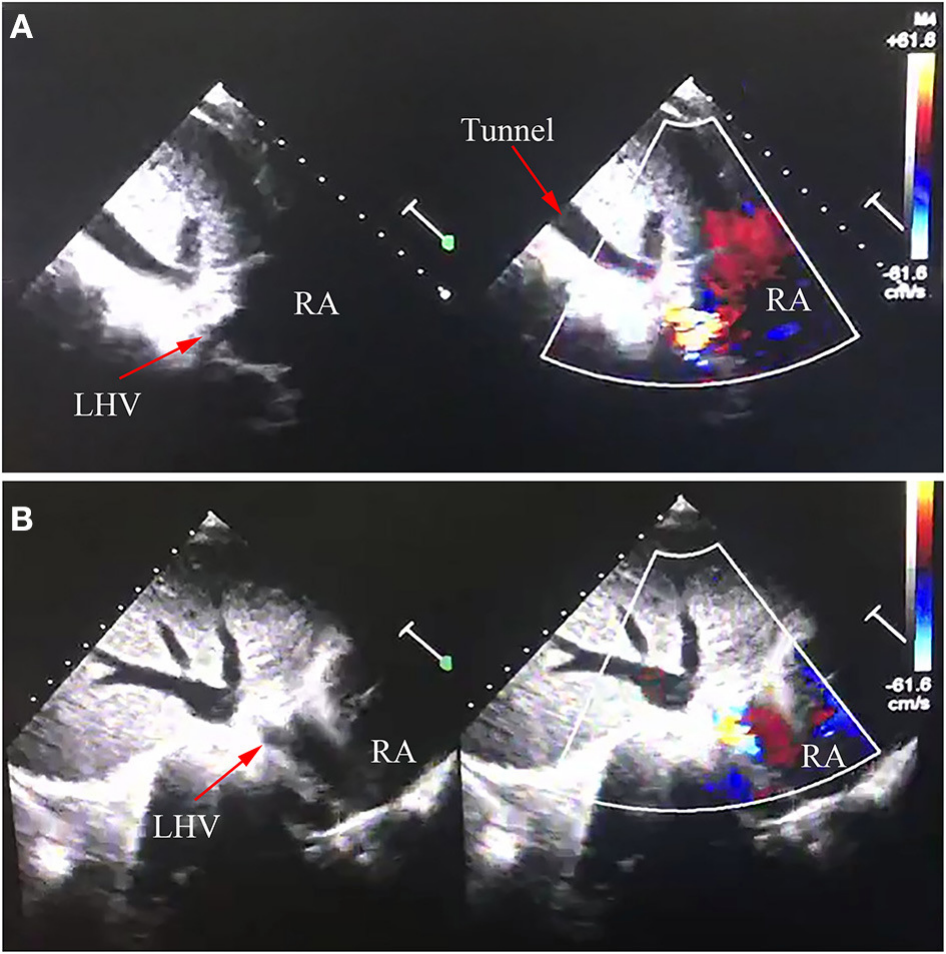
Echocardiography image of anomalous left hepatic venous connection. LHV, left hepatic vein; RA, right atrium. **(A,B)** Echocardiography image confirmed LHV came into the RA.

**Figure 2:**
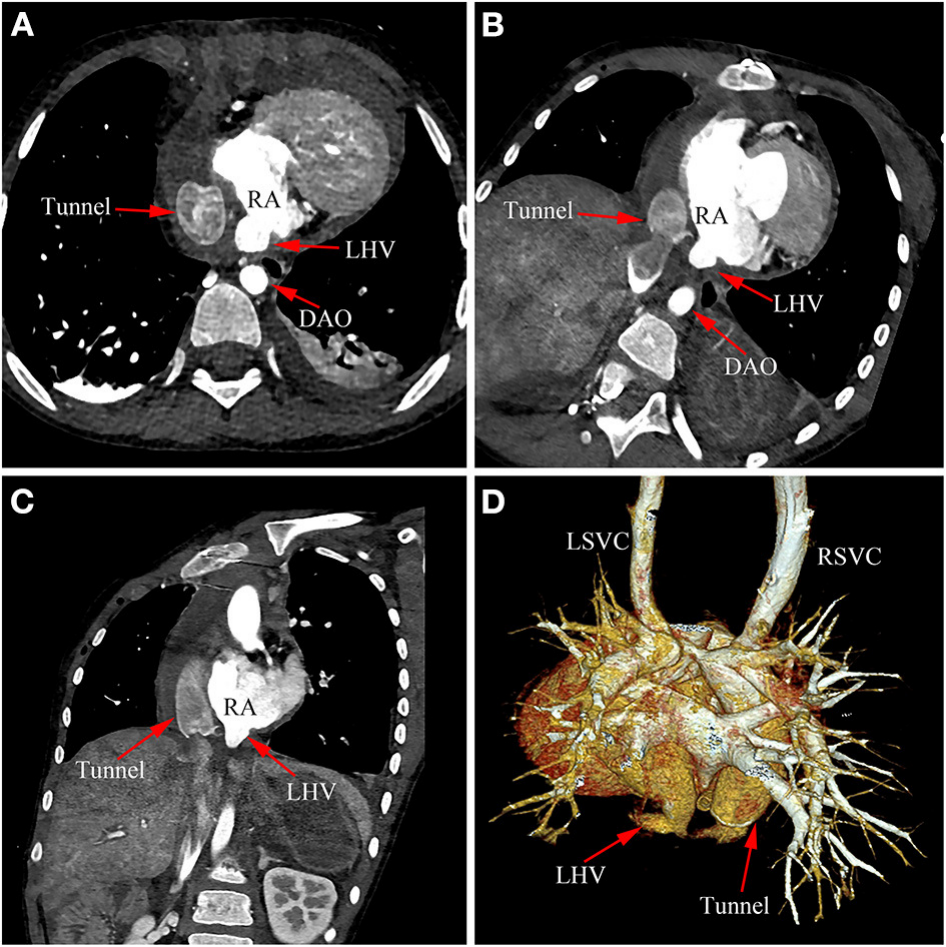
Cardiac CT image of anomalous left hepatic venous connection. **(A–D)** Postoperative CT image confirmed LHV came into the right atrium. LHV, left hepatic vein; DAO, descending aorta; RA, right atrium; RSVC, right superior vena cava; LSVC, left superior vena cava.

A 7-year-old Chinese boy about 1 year post bilateral SVC pulmonary anastomosis presented with severe cyanosis and was referred to our department. Physical examination revealed cyanosis with oxygen saturation in the upper and lower extremities of 78 and 76%, respectively. A recent local echocardiogram revealed the ventricular ejection fraction was 62%. The McGoon ratio was 1.8. A right heart catheterization in our hospital revealed well-developed left and right pulmonary arteries with a mean pulmonary artery pressure of 14 mmHg. Based on the clear diagnosis of echocardiogram in the local hospital, we did not perform cardiac computed tomography (CT) before operation. After extensive discussions with the patient and his family, an extracardiac total cavopulmonary connection (ETCPC) was scheduled. However, the patient showed an unexpected gradual decrease in blood oxygen saturation to 60–70% after the ETCPC operation. Emergency echocardiography and CT angiography confirmed that the external canal was unobstructed, with no thrombosis, but with abnormal influx of the left hepatic vein (LHV) into the atrium (Figures 1A,B; 2A–C, arrows), ETCPC (Figures 2C,D, arrows), and bilateral Glenn shunt (Figure 2D). The surgical correction was undoubtedly performed because the LHV was heterotopically drained into the atrium resulting in a right-to-left shunt, which led to a decrease in oxygen saturation. Subsequently, the patient was referred to the intra-atrial tunnel. The intraoperative finding was that the connective part of the LHV was parallel to the IVC and converged to the atrium at the left posterior sides. The patient was removed from tracheal intubation uneventfully on postoperative day 2 with the arterial oxygen saturation level returning to 98%. He is in good condition, and no related complications have been detected during the early period of following up.

## Discussion and Conclusions

An anomalous left hepatic venous connection is an extremely rare cardiac malformation, and left hepatic venous route abnormalities not associated with other cardiac lesions do not require surgical treatment because they are physiologically benign. However, when venous route abnormalities exist with associated cardiac lesions, the conduct of the cardiac surgical repair must accommodate the abnormal venous anatomy, especially in total cavopulmonary connection patients.

Physical examination is neither sensitive nor specific for the diagnosis ASVC. The chest radiograph and electrocardiogram may be normal, consistent with left- and/or right-sided cardiac enlargement or characteristic of any dominant associated cardiac malformation. ASVC can be diagnosed by a number of methods. Unfortunately, even in the current era of modern echocardiography and CT angiography despite many clues, the diagnosis is often delayed (5).

Echocardiography (transthoracic and/or transesophageal) is an extremely specific and highly sensitive method for making the diagnosis of congenital cardiovascular or cardiac abnormalities (6, 7). However, sometimes the diagnostic results or treatment effects are closely related to the operator's experience (8), especially for complex and rare congenital cardiovascular abnormalities. CT allows a non-invasive accurate diagnosis, depicting a three-dimensional assessment of the anatomic relations between the venous anomalies and adjacent structures, and it provides sectional views of cardiac structures from various angles. In the current era, non-invasive imaging, such as echocardiography and cardiac CT, has largely replaced cardiac angiography to establish the cardiac anatomy of patients with congenital heart disease. However, cardiac catheterization and angiography may need to be performed to supplement or clarify anatomical information obtained by non-invasive methods or to document hemodynamics, especially in patients with heterotaxy syndrome who may need to undergo staged Fontan palliation.

Bidirectional cavopulmonary and total cavopulmonary connections are now commonly performed in patients with a variety of forms of complex functionally single ventricle. When the systemic venous return is normal, a staged approach to total cavopulmonary connection is undertaken in the usual manner. Many of these patients, however, have anomalies of systemic and pulmonary venous return. While the standard extracardiac tunnel technique may be used under certain circumstances, it is sometimes necessary to resort to an intracardiac tube graft to avoid abnormal venous shunt.

This illustrative report highlights the essence of improving the preoperative accurate diagnosis to avoid unplanned reoperation in China, especially for the remote rural areas of eastern countries where the level of health care and services is relatively backward. Failure to identify anomalous LHV connection, in this case, will delay effective treatment past the optimal treatment time.

## Data Availability Statement

The original contributions presented in the study are included in the article/supplementary material, further inquiries can be directed to the corresponding author/s.

## Ethics Statement

The studies involving human participants were reviewed and approved by the Ethics Committee of Xiangya Hospital of Central South University, Changsha, China. The patients/participants provided their written informed consent to participate in this study. Written informed consent was obtained from the individual(s) for the publication of any potentially identifiable images or data included in this article. Written informed consent to participate in this study was provided by the participants' legal guardian/next of kin. Written informed consent was obtained from the individual(s), and minor(s)' legal guardian/next of kin, for the publication of any potentially identifiable images or data included in this article.

## Author Contributions

HB prepared the figures. HB and FL wrote the main manuscript text, collected, and checked the data. All authors reviewed the manuscript.

## Conflict of Interest

The authors declare that the research was conducted in the absence of any commercial or financial relationships that could be construed as a potential conflict of interest.

## Publisher's Note

All claims expressed in this article are solely those of the authors and do not necessarily represent those of their affiliated organizations, or those of the publisher, the editors and the reviewers. Any product that may be evaluated in this article, or claim that may be made by its manufacturer, is not guaranteed or endorsed by the publisher.
